# A total closed chest sheep model of cardiogenic shock by percutaneous intracoronary ethanol injection

**DOI:** 10.1038/s41598-020-68571-5

**Published:** 2020-07-24

**Authors:** Mario Rienzo, Julien Imbault, Younes El Boustani, Antoine Beurton, Carolina Carlos Sampedrano, Philippe Pasdois, Mathieu Pernot, Olivier Bernus, Michel Haïssaguerre, Thierry Couffinhal, Alexandre Ouattara

**Affiliations:** 1 Univ. Bordeaux, INSERM, UMR 1034, Biology of Cardiovascular Diseases, 33600 Pessac, France; 2 Department of Anaesthesia and Critical Care, Magellan Medico-Surgical Center, CHU Bordeaux, 33000 Bordeaux, France; 3 IHU LIRYC, Electrophysiology and Heart Modeling Institute, Fondation Bordeaux Université, Bordeaux, 33600 Pessac, France; 4 Univ. Bordeaux, INSERM, UMR 1045, Cardiothoracic Research Center of Bordeaux, 33600 Pessac, France; 5 Department of Cardiovascular Surgery, CHU Bordeaux, 33000 Bordeaux, France; 6 Department of Electrophysiology, CHU Bordeaux, 33000 Bordeaux, France; 7 CHU Bordeaux, Centre d’Exploration, de Prévention et de Traitement de l’Athérosclérose (CEPTA), 33000 Bordeaux, France; 8 Department of Anaesthesia and Critical Care, Magellan Medico-Surgical Centre, Bordeaux University Hospital, Av. Magellan, 33600 Pessac, France

**Keywords:** Cardiac device therapy, Acute coronary syndromes, Heart failure, Metabolomics, Experimental models of disease, Preclinical research, Translational research

## Abstract

To develop a reproducible and stable closed chest model of ischemic cardiogenic shock in sheep, with high survival rate and potential insight into human pathology. We established a protocol for multi-step myocardial alcoholisation of the left anterior descending coronary artery by percutaneous ethanol injection. A thorough hemodynamic assessment was obtained by invasive and non-invasive monitoring devices. Repeated blood samples were obtained to determine haemoglobin and alcohol concentration, electrolytes, blood gas parameters and cardiac troponin I. After sacrifice, tissue was excised for quantification of infarction and histology. Cardiogenic shock was characterized by a significant decrease in mean arterial pressure (− 33%), cardiac output (− 29%), dP/d*t*_max_ (− 28%), carotid blood flow (− 22%), left ventricular fractional shortening (− 28%), and left ventricle end-systolic pressure–volume relationship (− 51%). Lactate and cardiac troponin I levels increased from 1.4 ± 0.2 to 4.9 ± 0.7 mmol/L (p = 0.001) and from 0.05 ± 0.02 to 14.74 ± 2.59 µg/L (p = 0.001), respectively. All haemodynamic changes were stable over a three-hour period with a 71% survival rate. The necrotic volume (n = 5) represented 24.0 ± 1.9% of total ventricular mass. No sham exhibited any variation under general anaesthesia. We described and characterized, for the first time, a stable, reproducible sheep model of cardiogenic shock obtained by percutaneous intracoronary ethanol administration.

## Introduction

Five to 10% of patients presenting with acute myocardial infarction (AMI) develop cardiogenic shock (CS). This population has a high mortality rate (40%–50%)^1^ , despite the improvement in survival due to primary reperfusion therapy, optimal pharmacological treatment, intra-aortic balloon pumping and the use of short-term mechanical circulatory support (STMCS)^1^. Insights into the pathophysiology of CS remain crucial to the development of new approaches, either pharmacological or mechanical, to reduce local and systemic effects of CS, and to appraise safety and efficacy of novel procedures prior to clinical translation. In this context, large animal models may provide a heuristic methodology to test pathophysiological hypotheses and new therapeutic procedures. These models should mimic human pathology, be reproducible, allow for control of the extent of ischemia, rely on minimally invasive techniques, require short preparation time and easy animal management, lead to impaired cardiac function and be cost-effective. Several models of myocardial infarction exist and are based on coronary artery ligation, coronary artery occlusion (by angiography balloon catheter or embolization), or intra-coronary alcoholisation. However, the current large animal models are suboptimal for several reasons, e.g. high mortality rate^2^, inconsistency in infarct size^3^ and the invasive approach itself^4^. Development of a consistent and stable model of CS, is needed to more fully evaluate the interplay between hemodynamic and heart mechanics on one hand and STMCS on the other. Moreover, the current models do not fully mimic the clinical scenario of CS induced by selective coronary occlusion for several reasons. First, systemic or selective coronary hypoxemia^5^ is responsible for a generalized diminished cardiac function due to the hibernating myocardial condition: in this setting, cellular mechanisms of adaptation are not the same than in distinct infarcted myocardium. Similar concerns should be taken into account in the case of global toxicity, induced by carbon monoxide^6^. The use of microspheres could overcome this difficulty^7^, but the injection in the left main coronary artery has a generalized effect on the left ventricle and is associated with a jeopardized cellular death in the whole myocardial wall thickness. Furthermore, the set-up of several studies requires STMCS activation soon after^6,7^ or even before^8^ CS induction in order to avoid premature death of animals, or an open-chest procedure for coronary ligatures^9^, which completely modifies the heart-pericardium and heart–lung interactions. In recent studies suggesting that early mechanical percutaneous support prior to revascularization might reduce the size of myocardial necrosis^10^, a condition of mild systemic hypo-perfusion is obtained rather than an effective hemodynamic CS.

The present study aimed to describe a reproducible closed chest model of acute CS due to myocardial infarction, mimicking the clinical setting and taking into account some specific aspects of human pathology, such as myocardial cell death, in a precise delineated coronary territory involving the whole myocardial wall thickness. This model was intended to be relatively stable in time, reproducible and have a high rate of survival after the induction of CS.

## Methods

A total of thirty-two female Dorset sheep were used in this study. Their mean body weight was 49.0 ± 1.5 kg and they were 35 ± 6 months old. All animals received humane care in compliance with “European Community Standards on the Care and Use of Laboratory Animals” published on September 2010 (Directive 2010/63/EU on the protection of animals used for scientific purposes). The ethical committee N°050 of the French Ministry for Research and Innovation approved the project on 25 of September 2017.

### Anaesthesia

Animals were fasted overnight with free access to water, and premedicated with intramuscular injection of ketamine (10–20 mg kg^−1^), acepromazine 0.1 mg kg^−1^ (5 mg mL^−1^) and buprenorphine (0.03 mL kg^−1^). Surface electrocardiographic leads were applied and a single lumen peripheral catheter (18 G) was then placed in a peripheral vein of the front limb. Propofol (2 mg kg^-1^) was given for induction of anaesthesia while its maintenance was ensured by isoflurane (inspired fraction 0.5 to 1.2%) and continuous infusion of midazolam (0.1 mg kg^−1^ h^−1^) after endotracheal intubation. Sufentanyl was administered for analgesia and animal comfort (0.15 µg kg^−1^ h^−1^). Anaesthesia depth was adjusted during animal preparation by monitoring vital signs and pupil position, then kept stable during the procedure. Myorelaxation was ensured by a bolus dose of rocuronium (0.6 mg kg^−1^) followed by a continuous intravenous infusion (0.3 mg kg^−1^ h^−1^). Volume controlled mechanical ventilation was used to maintain gas exchange and relied on an Aysis CS2 station (GE Healthcare SAS, Boston, MA, USA) set on controlled volume mode (V_t_: 8 mL kg^−1^, Peep: 6–8 cmH_2_0). The ventilation was adjusted to maintain an E_T_CO_2_ between 35 and 40 mmHg, and the FiO_2,_ initially set to 0.5, was titrated to keep the PaO_2_ over 80 mmHg. 8 mL kg^−1^ of balanced crystalloids solution (Ringer Lactate Viaflo, Baxter, Deerfield, IL, USA) was administrated hourly. If intravascular volume expansion was required, hydroxyethyl starch was administered. 20 mg kg^−1^ of cefuroxime was given at the beginning of the experiment followed by 10 mg kg^−1^ re-injection every 2 h. Heart rhythm irregularities were prevented by using continuous infusion of amiodarone (1 mg  kg^−1^ h^−1^), magnesium sulphate (35 mg kg^−1^ h^−1^) and xylocaine (5 mg kg^−1^ h^−1^). A unfractioned heparin bolus (100 UI kg^−1^) was given after neck incision, followed by a continuous intravenous infusion (100 UI kg^−1^ h^−1^). A three-lumen catheter (Arrow Medical Ltd, Kington, UK) placed in the left jugular vein was used for these purposes. Body temperature was monitored by a thermal probe and stabilized between 36 and 37 °C by an external heating system. Gastric and bladder tubes were inserted to empty respectively the stomach and monitor urine outflow.

### Haemodynamic monitoring

All animals were monitored as follows:

- An intracavitary ventricular electrocardiogram was obtained by Millar probe-associated electrodes (Millar Inc, Houston, TX, USA) and used to monitor heart rhythm and identify any abnormalities. Surface electrocardiogram was used to synchronize echocardiographic imaging.
- A 6-F arterial catheter was placed in an artery of the anterior limb for continuous arterial pressure monitoring. TrueWave transducers (Edwards Lifesciences Corp, Irvine, CA, USA) were used for this purpose.
- A Doppler probe (Transonic Systems Inc., Ithaca, NY, USA) was positioned around the left carotid artery.
- A CCOmbo pulmonary artery catheter (Edwards Lifescience) connected to a Vigilance monitor (Edwards Lifescience) was inserted in the left jugular vein through a 9-F introducer allowing for continuous monitoring of pulmonary and central venous pressures, cardiac index by thermodilution, venous oxygen saturation (SvO_2_) and core body temperature.
- A conductance catheter (Millar Inc) with 7-mm-spaced segments was inserted through an 8-F introducer in the right carotid artery. The probe was positioned in the left ventricle to collect ventricular pressures and volumes. The maximal positive (dP/d*t*_max_) and negative (dP/d*t*_min_) left ventricular pressure derivatives were calculated from the left ventricular pressure signal. The probe position was checked with radioscopy, transthoracic echocardiography and analysis of the collected signals. Volume calibration of the Millar conductance catheter was obtained by calculation of parallel conductance after distal pulmonary artery injection of a 15%-hypertonic saline solution. A 9-F introduction kit was placed in the right femoral vein and used to introduce a tip-ballooned 8F Fogarty catheter (Edwards Lifescience) up to the inferior vena cava junction with the right atrium, in order to control venous blood flow and allow end-systolic pressure–volume relationship calculation (ESPVR or maximal elastance or E_max_). During dynamic manoeuvres, a respiratory pause was performed to limit the cardio-respiratory interactions induced by mechanical ventilation.

### Data acquisition

A 16-bits Powerlab acquisition system (ADInstruments Ltd, Oxford, UK) was used for data collection and Labchart 8 (ADInstruments Ltd) for data analysis. Systems and captors were calibrated at the beginning of each experiment. A drift of the signals from the conductance catheter was researched before each point of intervention.

### Echocardiography

Transthoracic echocardiographic examination (Vivid E95 Vet, GE, Boston, MA, USA) was performed concomitant to hemodynamic data collection at each point of intervention. Loops were recorded and a blinded-analysis was conducted. In the parasternal mid-papillary short axis plane, endocardial borders were traced in end-diastolic and end-systolic frames: LV end-diastolic (LVEDA) and end-systolic (LVESA) areas were used to calculate the fractional area change (FAC = [LVEDA − LVESA)/LVEDA] × 100).

### Blood gas and electrolytes measurements

At each haemodynamic reading, blood samples were simultaneously obtained via the peripheral and pulmonary arterial catheters for blood gas analysis with lactate (Handheld Blood Analyzer iSTAT-1, Abbott Point of Care Inc., Princeton, NJ, USA). Blood samples were also taken to determine concentration of haemoglobin (UniCel DxH 800 Coulter Cellular Analysis System, Beckman Coulter, Inc., Brea, CA, USA), cardiac troponin I by immuno-assay, ethanol and key electrolytes (Na^+^, K^+^, Cl^-^, Ca^2+^) (AU2700 chemistry analyzer and DXI600 Access Immuno-assay system, Beckman Coulter) before infarct induction and prior to sacrifice.

### Cardiogenic shock induction

**Figure 1:**
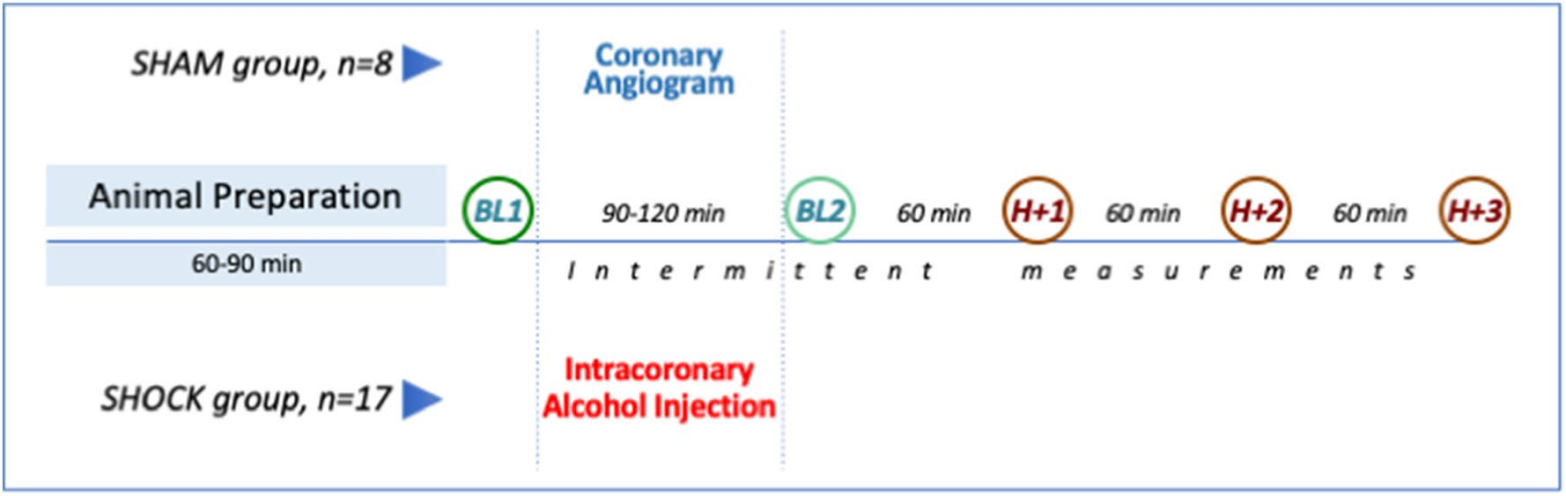
Study protocol. BL1 = baseline 1; BL2 = Baseline 2; H + 1 = 60 min after BL2; H + 2 = 120 min after BL2; H + 3 = 180 min after BL2.

As previously reported, an intra-coronary injection of ethanol was used to induce acute myocardial infarction^11^. We modified the technique by performing a sequential, multi-step myocardial “alcoholisation” procedure to obtain hemodynamically stable CS. An 8-F introduction kit was inserted in the right femoral artery and allowed the positioning of a 6F JL-guide catheter in the ostium of the main left coronary artery; after which a coronary angiography was performed. According to the distribution and segmentation of the left anterior descending coronary artery (LAD), an over-the-wire OPT 12 mm balloon catheter (Emerge OTW, Boston Scientific, Marlborough, MA, USA) was placed through a guide wire at different sites, progressing from the most distal to the most proximal. At each site, a sufficient volume of 99.9% ethyl alcohol was injected in order to completely block the blood flow distal to balloon inflation. Complete occlusion of the coronary artery was defined as TIMI (thrombolysis in myocardial infarction) flow ≤ 1 after alcohol injection. The injected alcohol volume depended on distal flow exclusion and rhythm disorder manifestations. The number of injected sites depended on the systemic hemodynamic consequences of necrosis extension. In accordance with previous animal models^5,8,9,12^, CS obtention was defined as a drop of more than 30% in mean arterial pressure and/or cardiac index associated to a rise of lactate concentration above 2.5 mmol L^−1^ in the hour following the last alcoholisation. Twenty-four animals underwent intracoronary alcoholisation (*SHOCK group*) and eight sham animals underwent interventricular coronary artery catheterization without any alcoholisation (*SHAM group*). A complete set of haemodynamic data (including standard hemodynamic, conductance catheter, echocardiographic derived parameters) was first collected after a 10 min stabilization period following the complete animal technical conditioning (Baseline 1), thirty minutes after CS constitution or one hour later for the SHAM group (Baseline 2) and every hour during a three-hour period (Fig. 1). Animal were sacrificed by intravenous injection of a lethal dose of sodium pentobarbital (≥ 100 mg/kg) before proceeding to sternotomy and heart explantation.

### Myocardial necrosis quantification

A sternotomy was performed at the end of experimentation. An aortic supra-coronary clamp was placed and Evans blue dye injected at the aortic root to colour the perfused cardiac tissues. The heart was then extracted and sectioned perpendicular to the main axis, to obtain 1 cm-thick sections. The sections were weighed, bathed for 10 min in a 2,3,5-triphenyl tetrazolium chloride (TTC) solution to discriminate infarcted and the area-at-risk (AAR). Both sides of each section were photographed and analysed using NIH Image J software to obtain a planimetric quantification of the infarcted area, expressed as a percentage of the left ventricle AAR and total ventricular mass; values for all sections from each heart were averaged.

### Myocardial histopathology

One section per animal was used for histologic and protein analyses. Biopsies of healthy and infarcted myocardium were processed for the following analyses:

- Optical histology-specimens were fixed in 10% formalin, cut and stained with haematoxylin–eosin (H/E) or Masson’s trichrome (M/T), after which they were examined for pathological changes in the infarcted myocardium and alcohol-injured LAD region.
- Electron Microscopy-specimens were snap frozen directly in optimum cutting temperature (OCT) compound; after being frozen at − 80 °C, samples were cut into sections measuring 20 nm and analysed for tissue oedema, mitochondrial morphology and disruption of the intercellular tight-junctions in order to evaluate the effects of ventricular wall stress.

### Statistical analysis

Results are expressed as mean ± SEM. A student’s t-test was used to compare two mean values. An ANOVA was performed for comparison of multiple means and post-hoc test analysis was performed using a Newman–Keuls test. A p-value < 0.05 was required to reject the null hypothesis.

## Results

### Closed-chest ischemic cardiogenic shock model

Twenty-four animals underwent CS induction (*SHOCK*), according to our protocol, and seven expired prior to completion of the protocol due to refractory rhythmic complications, thus the procedure had a 71% survival rate. Among the remaining 17 *SHOCK* animals, 5 required a very low dose (mean dose ≤ 0.01 µg kg^−1^ min^−1^) continuous epinephrine infusion as a rescue therapy after reversal of ventricular fibrillation or tachycardia to maintain systolic arterial pressure ≥ 55 mmHg, after the induction of CS. The mean intracoronary alcohol volume necessary to obtain CS was 6.1 ± 0.8 mL. The mean time between beginning of the alcoholisation procedure and shock instauration was 84 ± 8 min. There were no significant differences in electrolytes concentration between the SHOCK and SHAM groups (Na^+^ = 140 ± 2 mmol L^−1^; Cl^−^ = 105 ± 4 mmol L^−1^; K^+^ = 2.7 ± 0.3 mmol L^−1^ and Ca^2+^ = 2.17 ± 0.12 mmol L^−1^). Ethanol concentrations were undetectable in blood obtained from animals in which CS was induced.

**Figure 2:**
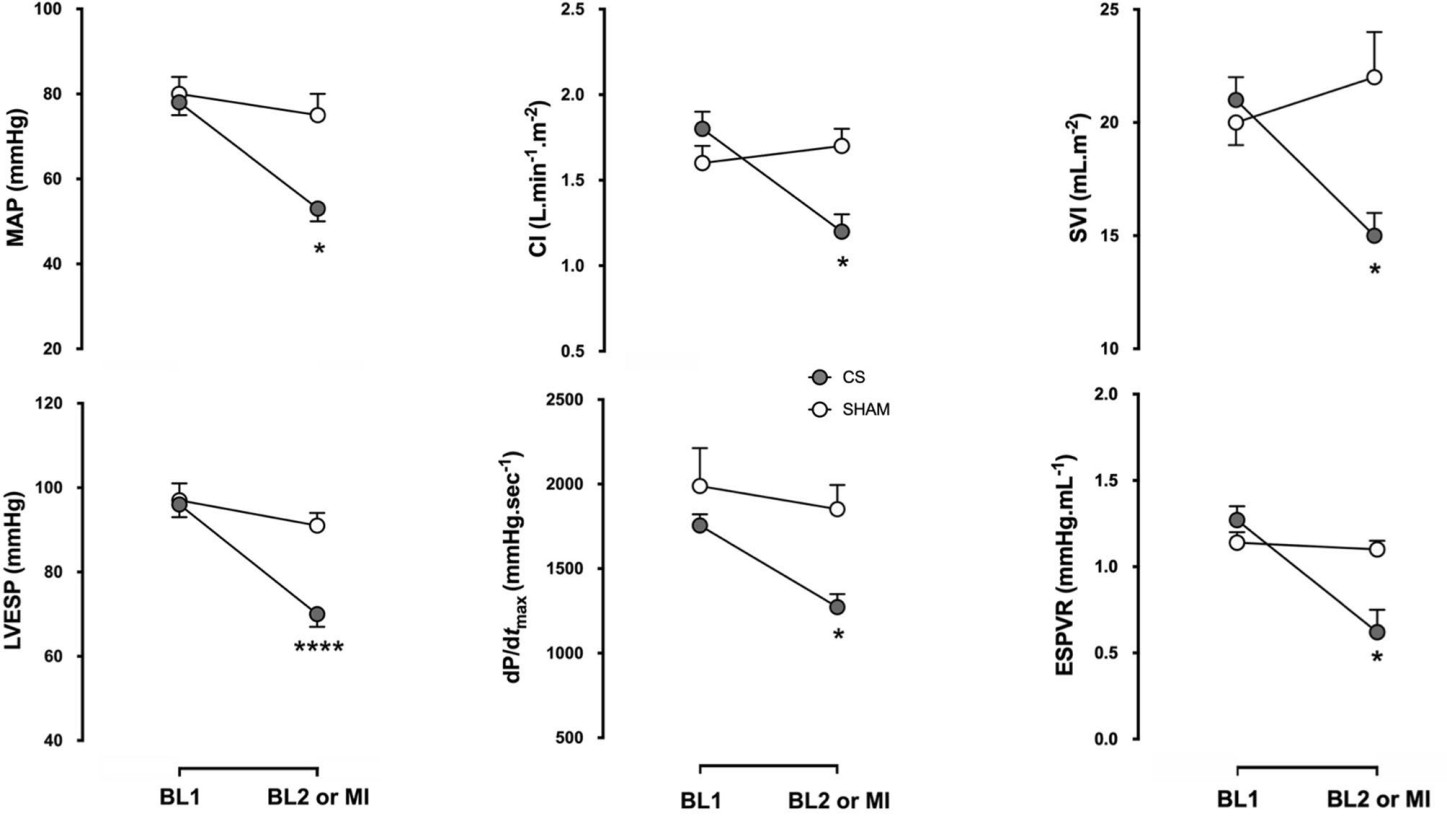
Changes in haemodynamic parameters between baseline 1 (BL1) and baseline 2 (BL2) in *SHAM* (open circles) and *SHOCK* (closed circles) groups are presented as mean ± SEM. Representative data from n = 17 CS and n = 8 SHAM animals is presented. A paired Sudent's t test was used to compare two mean values. *p<0.05 versus BL1. *MAP* mean arterial pressure, *CI* cardiac index, *SVI *Stroke volume index, *LVESP* Left ventricular end-systolic pressure, *dP/dt*_max_ maximal positive left ventricular pressure derivative, *ESPVR* End-systolic pressure-volume relationship, *MI* myocardial infarction.

The haemodynamic profile of CS is summarized in Table 1. It was characterized by a significant decrease in mean arterial pressure (− 33%), cardiac output (− 29%), end-systolic left ventricular pressure (− 26%) and dP/d*t*_max_ (− 28%). A greater decrease in the dP/d*t*_min_ was observed (− 55%). Carotid blood flow (− 22%) and left ventricular fractional shortening (− 28%) also decreased, while left ventricle tele-diastolic pressure increased (+ 38%). Due to the progressive complexity of our hemodynamic monitoring, Millar-derived parameters were not available for all animals (*SHOCK* n = 5, *SHAM* n = 6). The Millar-derived parameter ESPVR was significantly reduced (− 51%). A significant decrease in SvO_2_ was also observed. Lactate and cardiac troponin I levels increased from 1.4 ± 0.2 to 4.9 ± 0.7 mmol/L (p = 0.001) and from 0.05 ± 0.02 to 14.74 ± 2.59 µg/L (p = 0.001), respectively. These variations remained stable in *SHOCK* animals during the follow-up period while *SHAM* animals (n = 8) did not exhibit any variation at any timepoint (Tables 2, 3 and Fig. 2).

**Table 1:** Haemodynamic profile of cardiogenic shock (baseline 2) induced by ethanol.

	Baseline 1	Baseline 2	p value
Heart rate, bpm	85 ± 4	78 ± 3	0.04
Systolic arterial pressure, mmHg	98 ± 3	69 ± 2	< 0.01
Mean arterial pressure, mmHg	79 ± 13	53 ± 11	< 0.01
Diastolic arterial pressure, mmHg	68 ± 14	44 ± 12	< 0.01
LVESP, mmHg	96 ± 3	70 ± 3	< 0.01
LVEDP, mmHg	8 ± 1	11 ± 1	< 0.01
LV dP/dt_max_, mmHg s^−1^ (n = 11)	1,755 ± 66	1,272 ± 77	< 0.01
LV dP/dt_min_, mmHg s^−1^ (n = 11)	− 1,858 ± 124	− 833 ± 65	< 0.01
Right atrial pressure, mmHg	9 ± 1	12 ± 1	0.01
Mean pulmonary arterial pressure, mmHg	19 ± 1	21 ± 1	0.14
Cardiac index, L min^−1.^m^−2^	1.8 ± 0.1	1.2 ± 0.1	< 0.01
Stroke volume index, mL m^−2^	21 ± 1	15 ± 1	< 0.01
LV SW, mmHg mL (n = 6)	2,587 ± 282	791 ± 132	< 0.01
ESPVR, mmHg mL^−1^ (n = 5)	1.27 ± 0.06	0.62 ± 0.13	0.02
Fractional area change, % (n = 9)	49 ± 7	35 ± 5	< 0.01
Carotid blood flow, mL min^−1^ (n = 13)	404 ± 31	294 ± 21	< 0.01
SvO_2_, %	82 ± 1	73 ± 2	< 0.01

Data from n = 17 independent experiments (if not otherwise mentioned), expressed as mean ± SE. p value refers to comparison between baseline 1 and 2 by using a paired student’s t-test.

*LVESP* left ventricular end-systolic pressure, *LVEDP* left ventricular end-diastolic pressure, *dP/dt* first derivative of left ventricular pressure; *LVSW* Left ventricular stroke work; *ESPVR* end-systolic pressure volume relationship, *SvO*_*2*_ mixed venous saturation of oxygen.

**Table 2:** Haemodynamic parameters during three consecutive hours from baseline 2 (cardiogenic shock).

	Baseline 2	H + 60	H + 120 min	H + 180 min	Intragroup comparison (time effect)	Intergroup comparison (group effect)
**HR, bpm**						
SHAM	82 ± 5	81 ± 5	83 ± 5	82 ± 4	0.99	0.02
SHOCK	78 ± 3	75 ± 4	73 ± 5	69 ± 4	0.44	
**CVP, mmHg**						
SHAM	9 ± 1	9 ± 1	11 ± 1	11 ± 1	0.31	< 0.01
SHOCK	12 ± 1	12 ± 1	13 ± 1	13 ± 1	0.46	
**MPAP, mmHg**						
SHAM	20 ± 1	22 ± 1	22 ± 2	23 ± 1	0.43	0.59
SHOCK	21 ± 1	20 ± 1	21 ± 1	22 ± 1	0.39	
**LVESP, mmHg**						
SHAM	91 ± 3	89 ± 3	93 ± 4	90 ± 4	0.78	< 0.01
SHOCK	70 ± 3	64 ± 2	63 ± 3	62 ± 2	0.13	
**LVEDP, mmHg**						
SHAM	10 ± 6	11 ± 6	12 ± 4	12 ± 4	0.84	0.21
SHOCK	11 ± 1	12 ± 1	14 ± 2	14 ± 2	0.48	
**MAP, mmHg**						
SHAM	75 ± 5	72 ± 3	77 ± 3	73 ± 3	0.80	< 0.01
SHOCK	53 ± 3	48 ± 2	47 ± 2	45 ± 2*	0.04	
**dP/dt**_**max**_**, mmHg s**^**−1**^						
SHAM	1,851 ± 143	1,799 ± 146	1,966 ± 188	1,858 ± 246	0.93	< 0.01
SHOCK (n = 11)	1,272 ± 77	1,144 ± 57	1,127 ± 64	1,079 ± 98	0.52	
**dP/dt**_**min**_**, mmHg s**^**−1**^						
SHAM	− 1,860 ± 277	1,653 ± 155	− 1,770 ± 153	− 1,613 ± 157	0.80	< 0.01
SHOCK (n = 11)	− 833 ± 65	− 785 ± 44	− 736 ± 52	− 674 ± 74	0.58	
**CI, L min**^**−1**^						
SHAM	1.7 ± 0.1	1.7 ± 0.1	1.7 ± 0.1	1.7 ± 0.1	0.95	< 0.01
SHOCK	1.2 ± 0.1	1.2 ± 0.1	1.1 ± 0.1	1.0 ± 0.1	0.55	
**SVI, mL m**^**−2**^						
SHAM	22 ± 2	22 ± 1	22 ± 1	22 ± 1	0.99	< 0.01
SHOCK (n = 15)	15 ± 1	14 ± 1	14 ± 1	15 ± 1	0.53	
**SvO**_**2**_**, %**						
SHAM	83 ± 2	82 ± 3	82 ± 2	82 ± 2	0.99	< 0.01
SHOCK (n = 15)	73 ± 2	72 ± 12	67 ± 2	64 ± 2*	0.03	
**CBF, mL min**^**−1**^						
SHAM	419 ± 42	394 ± 40	380 ± 43	366 ± 47	0.84	< 0.01
SHOCK (n = 13)	293 ± 21	277 ± 15	269 ± 21	261 ± 25	0.69	

Data from n = 8 SHAM and n = 17 SHOCK independent experiments (if not otherwise mentioned), expressed as mean ± SE. Two-way ANOVA was performed for comparison of multiple means. * p<0.05 versus baseline 2.

*HR* heart rate, *CVP* central venous pressure, *MPAP* mean pulmonary arterial pressure, *LVESP* left ventricular end systolic pressure, *LVEDP* left ventricular end diastolic pressure, *MAP* mean arterial pressure, *dP/dt* first derivative of left ventricular pressure, *CI* cardiac index, *SVI* stroke volume index, *SvO*_*2*_ mixed venous oxygen saturation, *CBF* carotid blood flow.

**Table 3:** Biological parameters during three consecutive hours from baseline 2 (Cardiogenic Shock).

	Baseline 2	H + 60	H + 120 min	H + 180 min	Intragroup comparison (time effect)	Intergroup comparison (group effect)
**pH**						
SHAM	7.49 ± 0.02	7.48 ± 0.01	7.49 ± 0.01	7.48 ± 0.01	0.97	0.03
SHOCK	7.44 ± 0.02	7.45 ± 0.03	7.46 ± 0.03	7.42 ± 0.03	0.37	
**PCO**_**2**_**, kPA**						
SHAM	5.0 ± 0.2	5.0 ± 0.2	5.2 ± 0.2	5.1 ± 0.2	0.92	0.21
SHOCK	5.0 ± 0.2	4.9 ± 0.2	4.4 ± 0.3	5.0 ± 0.1	0.41	
**PO**_**2**_**, kPA**						
SHAM	28 ± 3	27 ± 3	27 ± 3	25 ± 3	0.88	0.21
SHOCK	28 ± 2	26 ± 2	22 ± 2	20 ± 2	0.08	
**HCO**_**3**_**, mmol L**^**−1**^						
SHAM	29.6 ± 1.0	28.1 ± 1.1	29.8 ± 1.3	28.6 ± 1.0	0.71	< 0.01
SHOCK	26.2 ± 1.0	25.7 ± 1.1	23.4 ± 1.9	24.8 ± 1.4	0.55	
**Lactates, mmol L**^**−1**^						
SHAM	1.3 ± 0.1	1.1 ± 0.1	1.1 ± 0.1	0.9 ± 0.1	0.41	< 0.01
SHOCK	3.6 ± 0.4	4.9 ± 0.7	4.8 ± 0.7	5.2 ± 1.0	0.42	

Data from n = 8 SHAM and n = 17 SHOCK independent experiments, expressed as mean ± SE. Two-way ANOVA was performed for comparison of multiple means.

### Echocardiography parameters

All *SHOCK* animals uniformly showed apico-antero-septal akinesis associated with a decrease in the fractional area change (Table 1).

### Morphometric and histological analysis

**Figure 3:**
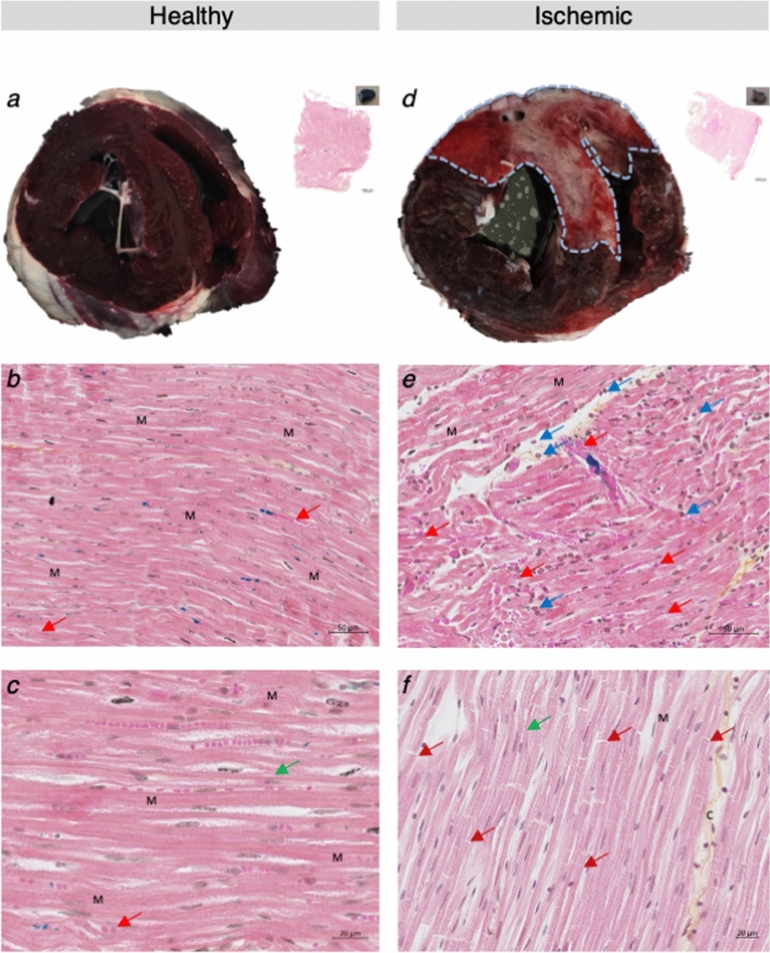
Macroscopic and microscopic aspect of healthy and infarcted myocardium from two representative animals, one CS and one SHAM. Top of figure: macroscopic aspect of healthy (**a**) and infarcted (**d**) myocardium after Evans blue dye and 2,3,5-triphenyl tetrazolium chloride coloration. The dotted blue line delimits the ischemic territory. Bottom of figure: microscopic aspect of healthy (**b**, **c**) and infarcted myocardium (**e**, **f**). (**b**, **e**) scale: 50 μm; (**c**, **f**) scale: 20 μm. Red arrows: red blood cells. Blue arrows: leucocytes. Green arrows: sarcomeres. Dark red arrows: contraction bands. C: collagen. M: myocardial cells.

**Figure 4:**
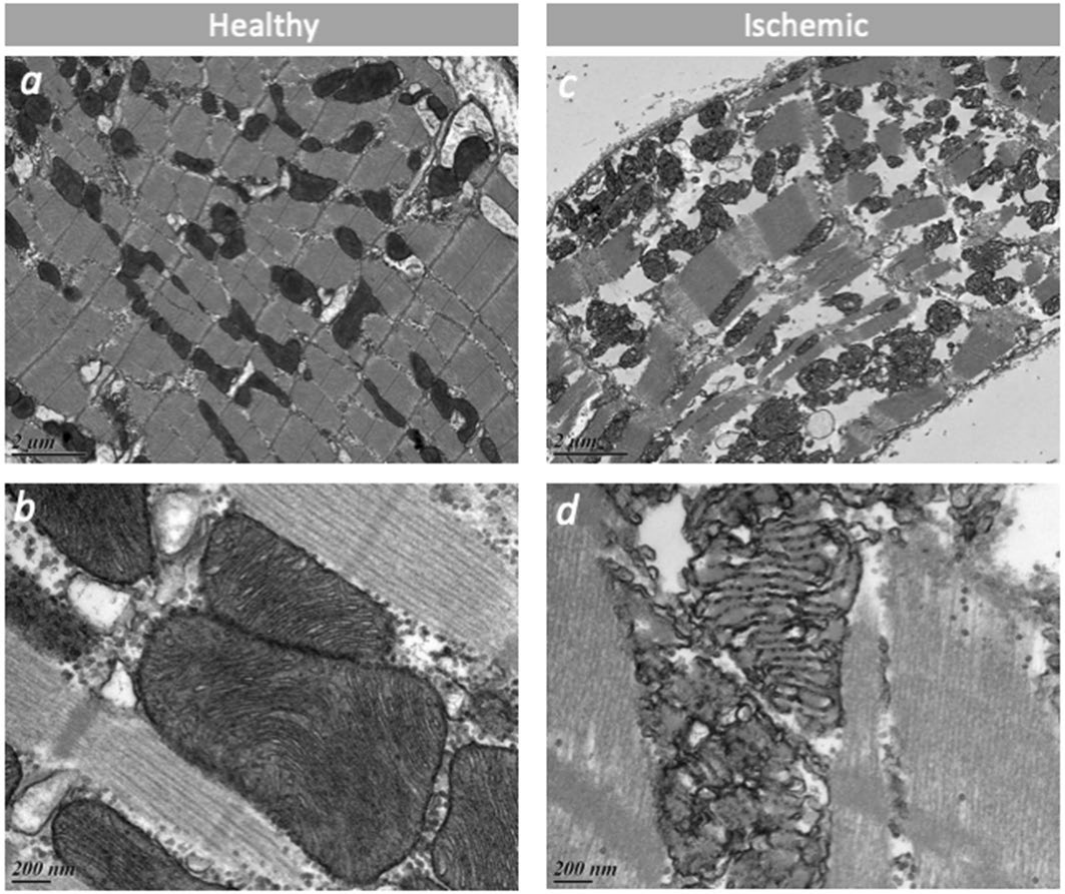
Electron microscopy of healthy (left panel: **a**, **b**) and infarcted (right panel: **c**, **d**) myocardium from two representative animals, one CS and one SHAM. ‘**a**’ and ‘**c**’ scale: 2 μm; ‘**b**’ and ‘**d**’ scale: 200 nm. In the healthy non-ischemic zone, mitochondria are aligned along the normally structured muscle fibres (**a**), very dense, not swollen and the inner membrane folding (Cristae) are well preserved (**b**). In the ischemic zone, the mitochondrial network is disorganized (**c**), mitochondria are swollen, the inner membrane folding (Cristae) is completely altered (**d**).

*SHOCK* animals (n = 5) uniformly showed transmural perfusion defects in the apico-antero-septal wall; the necrotic volume and overall ischemic region (AAR) represented respectively 24.0 ± 1.9% and 43.9 ± 3.8% of LV volume, yielding an ‘infarct to AAR ratio’ of 0.55 ± 0.03 (Fig. 3). Pathological observations (in two animals) with H/E staining demonstrated loss of cross striations, contraction bands, some oedema with haemorrhage and neutrophil infiltrate, which are all signs of lesions associated with myocardial cellular degeneration (Fig. 3). Analysis by electron microscopy showed clear differences between *SHAM* (n = 1) and *SHOCK* (n = 1) animals. In samples from the *SHAM* animal the mitochondria were very dense and not swollen, and the inner membrane folding (Cristae) was well preserved (Fig. 4a, b). On the contrary, in samples from the *SHOCK* animal the mitochondria were swollen and broken with the inner membrane folding (Cristae) completely altered (Fig. 4c, d).

## Discussion

We described here, for the first time, a reproductible CS model obtained in sheep by percutaneous intracoronary ethanol injection that demonstrates a significant decrease in mean arterial pressure (− 33%), cardiac output (− 29%), dP/d*t*_max_ (− 28%), dP/d*t*_min_ (− 55%), carotid blood flow (− 22%), left ventricular fractional shortening (− 28%), left ventricle end-systolic pressure–volume relationship (− 51%) and remained stable over a three-hours period. Microscopic analysis revealed signs of myocardial cellular degeneration, *e.g.* loss of cross striations, contraction bands, some oedema with haemorrhage and neutrophil infiltrate; electron microscopy analysis revealed swollen mitochondria with the inner membrane folding completely altered in infarcted tissue.

### Large animal model

The use of large animal models in cardiac physiology offers some advantages, including clinical relevancy, the possibility to test delivery devices based on catheters and to study the dose–effect relationships^13^. Several characteristics make sheep a valuable species; unlike canine models and similar to humans, they do not sufficiently develop a collateral circulation network beneath the epicardium: as a result, it is easier to induce myocardial infarction in a reproducible manner, including the related issues of myocardial hibernation, myocardial stunning^14^, and LV aneurysm^15^. Moreover, unlike bovine models and similar to humans, sheep allow studies on angiogenesis^16^. Even if swine have a cardiac morphology very similar to that of humans^17^, they grow up relatively rapidly, which makes it harder to conduct long-term studies; furthermore, it is challenging to set up devices for thoracotomy and cardiopulmonary bypass in this species^18^.

### Closed chest model

The pioneer sheep models of antero-apical MI^19^ and postinfarction heart failure^20^ had a disadvantage in that they required a thoracotomy due to the variability of coronary arterial anatomy. Contrary to them, all closed-chest models have the advantage to profoundly limit the huge trauma of thoracotomy, thus respecting cardiac and whole-body physiology and favouring recovery. Percutaneous methods also show reliability and reproducibility, and are able to induce myocardial infarction with a pathophysiology mimicking that of myocardial ischemia in human clinical setting. However, they have inherent limitations. (1) In percutaneous models employing a microsphere embolization technique, the precise quantity of particles injected is required^14^, the volume and the anatomical location of myocardial necrosis cannot be selectively produced, thus rendering the extent of myocardial damage poorly predictable^21^; moreover, in some dog models, left ventricular function is acutely and severely depressed, leading to a high mortality, while survivors experience an almost complete recovery, partially due to the rich collateral circulation of the canine heart^22^. (2) The exploration of well-defined ischemia and related cardioprotective therapies are barely explored by models established with coronary thrombosis because of the variability of time needed for occlusive thrombus formation, and of the difficult control of duration and severity of ischemia^23^. (3) Models employing angioplasty balloon inflation and deflation are typically used to explore the myocardial reperfusion phenomenon, but they are burdened with high incidence of left ventricular fibrillation^24^. (4) When a metallic coil embolization technique is utilized, it is almost impossible to determine in advance the site of the arterial obstruction, which results in considerable variability of the ischemic areas sizes among different animal species^25^. (5) Gelatine sponge embolization procedure as well is complicated by high rate of ventricular fibrillation, ventricular tachycardia and mortality^2^. In our view, this totally closed-chest sheep CS model addresses and overcomes many of these limitations and importantly, it has a high survival rate of (71%). Although few authors specifically reported this criterion^26^, this high survival rate appears to be another strength of this model: we provide, as additional data, a table summarizing the main features of known cardiogenic shock animal models (Annexe 1).

### Hemodynamic profile of cardiogenic shock model

As expected, our closed-chest CS model is characterized by approximately a 30% decrease of systemic arterial pressure and cardiac output as well as inotropic parameters. As previously reported, myocardial infarction appears to affect more severely lusitropic properties^27^. Although our CS model was associated with a significant decrease in SvO_2_, it should be pointed out that the value remains relatively high in comparison to a previous reported model^12^. This difference may be explained by two reasons: (1) the fact that all our animals were maintained under general anaesthesia, including myorelaxant agents, which could be responsible for a profound decrease in oxygen consumption; (2) the high level of arterial oxygenation during the entirety of the experiment. However, a profound end-organ hypoperfusion state was confirmed by a significant increase in lactate value. More severe CS has been previously reported^7,8,12^, but most of these authors implanted a mechanical circulatory support device before the induction of CS or immediately after^7, 8^. In our model, heart rate was slightly but significantly lower after CS induction. Such a slight decrease is unlikely to be responsible for the drop in cardiac output, or the low systolic volume and consequently affected left ventricular ejection fraction. Most previously reported models of CS noted a compensatory tachycardia^8,28^, rather than bradycardia. However, our findings may be easily explained by the fact that at the beginning of the experiment, all animals received analgesia based on sufentanyl associated to a mixture of xylocaine, amiodarone and sulphate magnesium to prevent arrhythmic irregularities. These drugs are known to induce bradycardia and could have influenced the hemodynamic profile of our model^29–31^. These bradycardizing effects could have been accentuated by ischemic dysfunction of the sinus node. Finally, as we were unable to detect any alcohol plasma concentrations in animals, an inhibition of baroreflex bradycardia through GABA receptors by ethanol injection^32^ is highly unlikely. It should be noted that an absence of compensatory tachycardia has been widely reported by others developing animal model of CS^7,33,34^. Lastly, five animals received epinephrine. Because the mean dose was ≤ 0.01 µg kg^−1^ min^−1^, it is highly unlikely that a such dose could significantly modify the hemodynamic profile of cardiogenic shock in our model.

### Coronary alcoholisation

Intracoronary alcohol injection was firstly used to treat arrhythmias in patients suffering from chronic ischemic heart disease or myocarditis^35^, and to relieve the ventricular pressure gradient in symptomatic patients suffering from obstructive hypertrophic cardiomyopathy^36^. Inspired by these clinical settings, intracoronary alcohol injection has become a supplemental approach in animal models of ischemic myocardial dysfunction: under video-fluoroscopic guidance, an ethyl alcohol solution (at variable percentage) is injected in specific coronary vessels according to predetermined experimental goals. This method has proven to be simple and reproducible^37,38^ and, compared to other techniques, it enables the TIMI flow grade evaluation immediately after the injection and without removing the balloon device^13^.

### Alcohol concentration and Infarct Size

Previous findings have shown that solutions with slight ethyl alcohol concentration are not adequate to induce tissue damage^39^ and that the severity of myocardial damage tends to be greater with solutions that have a higher ethyl alcohol concentration (50 to 100%)^35,37^. The use of pure alcohol for coronary embolization in our experimental model allowed us to obtain homogeneous infarct sizes of 24 ± 4% of ventricular mass. These results differ from measures reported by a recent review paper^27^ in which infarct size measured by TTC staining is described as ranging from 30 to 60% of the total LV volume. We are not able to fully explain these discrepancies: studies cited by Linsdey^27^ vary in several ways (animal size, infarct induction, time elapsed from infarct induction until sacrifice) whereas studies describing alcohol concentration do not express infarct size as a percentage of total ventricular mass^35,37^.

### AAR/infarct size ratio

It has previously been demonstrated that infarct size strongly depends on the extension of the occluded coronary bed, rather than the anatomic occlusion site, regardless of the oxygen demand-supply balance^40^. Accordingly, although a variable number of regions of the LAD territory were injected with alcohol, we were able to obtain a homogeneous percentage of AAR and “infarct size to AAR ratio” (infarct to AAR volume ratio 0.55 ± 0.03).

### Histological effects of coronary alcoholisation

Differential effects of intracoronary alcohol injection on coronary vessels or myocardial tissue have been previously described^37^. As regards intraluminal response, a persistent and extensive thrombi constitution is prompted by high concentrations of alcohol, distally to the injection site^35^ due to sub-endothelium exposure. Progressively, thrombus is organized and calcified and a chronic total occlusion ensues^18^. As regards myocardial structure, myocardial necrosis occurs from 100% ethanol injection into the canine coronary circulation, either arterial or venous: it is focal or patchy and non-confluent, and involves more than half the thickness of the left ventricular wall^35^. Direct intra-myocardial alcohol injection induces myocardial necrosis without intraluminal thrombosi^41^.

If severe ischemia lasts more than 20- to 30-min, cardiomyocytes in the subendocardial area undergo irreversible changes ranging from sarcolemmal disruption to striking perturbations in mitochondrial architecture; typical mitochondrial ultrastructural alterations are represented by amorphous matrix densities and severe swelling. All cardiomyocytes showing these ultrastructural alterations cannot be rescued and they will constitute the infarct environment after death^42^. According to studies on reperfused infarction in sheep, it would seem that the mid-myocardium is more vulnerable to ischemic injury whereas the sub-endocardium is relatively resistant^43^.

Accordingly, we found thrombi in ischemic tissue and coagulative necrosis at an early stage. Optical microscopy revealed that the full myocardial thickness was involved in the ischemic process, with muscular fibres appearing to be hyper-contracted, while nuclei were still present because animals were sacrificed before their disappearance (18–24 h). Electron microscopic analysis revealed that the mitochondrial network was disorganized, mitochondria swollen, and the inner membrane folding completely altered*;* indicating that the mitochondria were broken and unable to provide any energetic supply for cell survival^44^.

### Correlation “AAR/infarct size ratio”: haemodynamic

The fall in coronary blood flow is immediately followed by myocardial ischemia, which implies myocardial anaerobic metabolism, fast ATP depletion, and metabolic waste amassing; the myocardial mechanism is severely altered and results in harsh systolic and diastolic dysfunction, and the ischemic muscle stretches instead of shortens during systole^45^. As ischemia progresses (less than 15 min), the myocardium turns into stunning and is not forthwith responsive to reperfusion^46^. Whenever injury continues, myocytes death leads to infarction^42,47^. Several clinical and experimental observations^48,49^ suggest that CS complicating myocardial infarction is accompanied by extensive myocardial injury and that at least 40% of left ventricular volume should be ischemic to induce CS. Nevertheless, in the clinical scenario, a severe degree of myocardial insult is not always necessary to cause CS^45^.

Although in our experimental model we observed homogeneous infarct sizes lower than those meant to induce CS, the overall ischemic region, including the AAR, represents 43.9 ± 3.8% of LV volume. This is consistent with the concept that myocardial dysfunction can develop even in the presence of viable tissue: the presumed mechanism underlying this phenomenon is myocardial stunning, i.e. a condition of ischemic injury without myocardial necrosis or cell death^46^. Moreover, previous studies showed that hemodynamic variables become abnormal when the area at risk is larger than 25%^50^ or the infarct size is between 12 and 13%^28^ (Annexe 1). Of course, the alternative evolution towards a complete myocardial recovery or a myocardial necrosis cannot be determined because stunning may persist for hours to weeks^47^. Accordingly, we speculate that by prolonging the post-induction period and delaying the sacrifice, more myocardial tissue in the AAR would have become necrotic.

### Study limitations

No model is devoid of flaws.

1. Myocardial infarction is defined as myocardial necrosis consequent to myocardial ischemia^51^. In our model, a chemical injury rather than ischemia was responsible of the heart damage. Although the mechanism is not the same, the histological appearance, both on optical and electron microscopy, and the left ventricular kinetic pattern of the affected area are quite similar to classical infarct by myocardial ischemia.
2. Very few specimens have been used for microscopy investigation: one *SHAM* and two *SHOCK* for each histological approach (optical and electron): nevertheless, all findings found in *SHOCK* animals are pathognomonic of infarcted cardiac tissue and not present in controls.
3. The use of an ischemia–reperfusion model is usually suggested in order to explore potential strategies prompting a reduction of acute myocardial infarct size^25^: our model exposes myocardial tissue to a rapid sequence of ischemia–reperfusion phases when the balloon catheter is progressively pulled from the distal to the final proximal site of ethyl alcohol injection. Nevertheless, the intracoronary thrombosis remains irreversible.
4. We used healthy animals, whereas human patients with acute ischemic heart failure belong a population exhibiting several risk factors, such as diabetes, hypertension, and atherosclerosis^11^; nevertheless, almost all animal models are confronted with this limitation.
5. Lastly, the observation time was limited to three consecutive hours. This study period may appear to be short but it is similar to those proposed by other author^8^. The main reason of our choice to limit the study period is practical. The preparation of the experimental setting and CS induction took approximately 4–5 h. Consequently, an observation time of only three consecutive hours was compatible with operatory room organization.

### Possible utility of our model for future research

As previously outlined, few large animal models of consistent and stable refractory cardiogenic shock are available for research purpose to-date, and the previously established models do not completely mimic the clinical scenario of CS induced by selective coronary occlusion. In our experimental model, we do not use coronary hypoxemia or hypercarbia thus minimizing the hibernating myocardial condition, and we are able to obtain homogeneous trans-mural myocardial damage without jeopardizing cellular death in a precise coronary artery territory. Furthermore, permanent coronary occlusion mimics the clinical situation of patients enduring acute myocardial without timely or successful reperfusion because of contraindications or logistic issues^52^. In such a specific context, our experimental set-up could lead to a better understanding of mechanical interaction between short-term mechanical circulatory support or LVAD and an acutely failing ventricle, thus allowing a precise tuning of flow and pressure generated by an assist device. The potential usefulness or harmfulness of vasoactive and inotropic drugs (calcium inhibitors, alpha-2-antagonists, sympathomimetics or other like levosimendan) in patients assisted with those devices might also be explored in this model. Moreover, the impact of ventricular support on early remodelling of non-ischemic territory could be more fully investigated, as well as the time-course evolution of the AAR in cases where it is not possible to reperfuse: while LV mechanical unloading prior to coronary revascularization may theoretically reduce infarct size by reducing the AAR^10^, no experimental investigation has been undertaken in the case of permanent coronary occlusion.

## Conclusions

We described and exhaustively characterized a unique, reproductible, homogenous and stable sheep model of CS obtained by percutaneous intracoronary ethanol administration. This technique grants trans-mural infarct formation with a clinical profile close to the human one. We think that our model may be appropriate to better understand the mechanical interaction between short-term mechanical circulatory support and/or LVAD and an acutely failing ventricle, the impact of ventricular support on early remodelling of non-ischemic territory, as well as the time-course evolution of AAR in the case of impossible reperfusion.

## Supplementary information

Supplementary information

## Data Availability

The datasets generated during and/or analysed during the current study are available from the corresponding author on reasonable request.
