# Supplementary material for: A total closed chest sheep model of cardiogenic shock by percutaneous intracoronary ethanol injection
Source: Sci Rep. 2020 Jul 24;10:12417. doi: 10.1038/s41598-020-68571-5 (PMC7381645; doi:10.1038/s41598-020-68571-5)
Supplement: Supplementary file 1 — Supplementary information [file 41598_2020_68571_MOESM1_ESM.docx]

**Annex 1. Literature review of large animal models of cardiogenic shock**

Title of Manuscript:

“A total closed chest sheep model of cardiogenic shock by percutaneous intracoronary ethanol injection”

Authors:

Mario Rienzo MD PhD, Julien Imbault MD MSc, Younes El Boustani MD, Antoine Beurton MD, Carolina Sanpedrano DVM, Philippe Pasdois PhD, Mathieu Pernot MD PhD, Olivier Bernus PhD, Michel Haïssaguerre, MD PhD, Thierry Couffinhal MD PhD, Alexandre Ouattara MD PhD

| **N°** | **Authors** | **Animals** | **Weight (kg)** | **CS induction** | **Thorax** | **Definition of CS** | **Observation time** | **Infarct**  **size** | **Feasibility** | **Survival rate** | **Comments** |
| --- | --- | --- | --- | --- | --- | --- | --- | --- | --- | --- | --- |
| 1 | Lluch et al. 1969 ^1^ | Dog | 17-22 | Cx embolization | closed | hypotension, tachycardia, severe oliguria to anuria | 5-48 hours | unknow | 76% | 80% |  |
| 2 | Feola et al. 1971 ^2^ | Dog | 15-25 | LAD ligation | open | unknown | 1-2 hours | unknow | 85% | 60% |  |
| 3 | Chandraratna et al. 1973 ^3^ | Dog | 15-25 | Cx embolization | closed | - 50% CO and MAP < 70 mmHg | unknow | unknow | 12.5% | 92.5% |  |
| 4 | Bleifeld et al. 1974 ^4^ | Dog | 18-40 | LAD ligation | open | - 30% SAP and -20% stroke volume and + 40% LVEDP | 3 hours | unknow | 50% | 100% | ligation + IABP vs IABP alone |
| 5 | Freye et al. 1974 ^5^ | Dog |  | Cx ligation | open | - 35% LV pressure, -50% CO, ST elevation and T-inversion | 2-3 hours | unknow | 75% | 75% |  |
| 6 | Romero et al. 1974 ^6^ | Dog | 15-22 | Coronary embolization | closed | - 50% CO and - 35% MAP | 3 hours | unknow |  |  | isolated lung perfusion technique |
| 7 | Weisse et al. 1974 ^7^ | Dog |  | LAD or Cx occlusion | closed | - 33% reduction in aortic systolic pressure and SAP < 90 mmHg | 2-3 hours | unknow | 40% | 20% |  |
| 8 | Bavaria et al. 1988 ^8^ | Sheep | 34 | Aortic clamp and ventricular fibrillation | open | unknown | 1-2 hours | unknow | 91.7% | 100% | ECLS before CS |
| 9 | Wouters et al. 1993 ^9^ | Dog | 25-32 | LAD occlusion | open | unknown | 4-16 hours | 12-13% | 100% | 41.6% | MAP very low but CO stable |
| 10 | Reilly et al. 1997 ^10^ | Swine | 15-25 | Pericardial tamponade | open | unknown | unknow | unknow | 100% | 100% | - 47% CO |
| 11 | Jin et al. 2000 ^11^ | Swine | 35-45 | Ventricular fibrillation + electric cardioversion | closed | Decrease in SAP and CO after resuscitation | 6 hours | unknow | 100% | 100% |  |
| 12 | Drakos et al. 2005 ^12^ | Swine | 50-60 | Coronary artery ligation and propranolol | open | - 60a% ortic BP and LVEDP > 20 mmHg | 80 min | unknow |  |  | centrifugal pump or centrifugal pump + IAPB. Both were implanted before CS |
| 13 | Götberg et al. 2010 ^13^ | Swine | 40-50 | LAD occlusion | closed | SAP < 90 mmHg more than 15 min | 4 hours | unknow | 64% | 80% |  |
| 14 | How et al. 2010 ^14^ | Swine | 30 | LAD embolization | closed | unknown | 2 hours | unknow |  |  |  |
| 15 | Kawashima et al. 2011 ^15^ | Dog | 20-24 | LAD ligation | open | unknown | 2-3 hours | unknow |  |  | ECLS and Impella |
| 16 | Andersson et al. 2012 ^16^ | Swine |  | LAD occlusion | closed | unknown | 4 hours | unknow |  |  | 6 hypothermia vs 6 normothermia. CO < 2.5 L/min |
| 17 | Brehm et al. 2014 ^17^ | Swine | 65-74 | Esmolol | closed | unknown | unknown | unknow |  | 100% | ECLS before Esmolol, MAP < 50 mmHg |
| 18 | Møller-Helgestad et al. 2014 ^18^ | Swine | 70 | LAD occlusion | closed | SAP < 100 mmHg and reduction of SvO2 | unknown | unknow |  | 94% | Impella vs IABP (cross-over then combined) |
| 19 | Stenberg et al. 2014 ^19^ | Swine | 46-52 | Coronary embolization | closed | - 30% CI and MAP < 65 mmHg or systolic < 90 mmHg and hypoperfusion | 10-16 hours | unknow | 33% | 94% |  |
| 20 | Zhu et al. 2014^20^ | Mini-Pig | 33-36 | Coronary ligation | open | unknown | 24 hours | unknow | 92% | 92% | ECLS before CS |
| 21 | Ostadal et al. 2015 ^21^ | Swine | 45 | Selected coronary perfusion by hypoxemic venous blood from ECLS | closed | SAP < 100mmHg + lactates > 2 mmol/L or SVO2 < 50% or NIRS < 50% | 90 min | unknow | 100% | 100% | ECLS before CS |
| 22 | Ostadal et al. 2016 ^22^ | Swine | 45 | Upper body hypoxia with low FiO2 and ECLS with high FiO2 on lower body | closed | LVEF < 30% and CO < 3.5 L/min | unknow | unknow | 100% | 100% | ECLS before CS |
| 23 | Beurton et al. 2016 ^23^ | Swine | 45-50 | LAD ligation | open | - 20% CO and MAP, lactates > 2.5 mmol/l | 5 hours | unknow | 60% | 60% |  |
| 24 | Koudoumas et al. 2017 ^24^ | Swine | 30-35 | Coronary ligation | open | - 45% CO | unknow | 35% of LV |  |  | 2 different biventricular infarct patterns |
| 25 | Vanhuyse et al. 2017 ^25^ | Swine | unknow | Coronary ligation | open | - 20% CI and MAP and lactates > 2.5 mmol/L | 8 hours | unknow | 100% | 100% | ECLS with normo- or hypothermia |
| 26 | Trivella et al. 2017 ^26^ | Swine | 29-48 | LAD occlusion | open | N/A | 4-5 hours | unknow | 100% | 60% | ECLS |
| 27 | Bari et al. 2018 ^27^ | Mini pig | unknown | Pericardial tamponade | laparotomy | N/A | 240min | unknow |  |  |  |
| 28 | Simoensen et al. 2018 ^28^ | Swine | 45-51 | Carbon monoxide poisoning | open | - 50% CO | 50 min | unknow | 100% | 58% | ECLS |
| 29 | Ostadal et al. 2018 ^29^ | Swine | 45 | Upper body hypoxia with low FiO2 and ECLS with high FiO2 on lower body | closed | LVEF <30% and CO <3.5 L/min | 60 min | unknow | 100% | 100% | ECMO before CS |
| 30 | Møller-Helgestad et al. 2019 ^30^ | Swine | 71-77 | LAD embolization | closed | CO ≤ 2L/min or/and SvO2 ≤ 35% | 2-3 hours | unknow | 100% | 80% | ECLS versus Impella CP |
| 31 | Ferrari et al. 2020 ^31^ | Sheep | unknown | Pulmonary artery embolization | closed | - 50% CO and < 2.5 L/min | 4-8 hours | unknow | 100% | 88% | Right ventricular failure |
| 32 | Udesen et al. 2020 ^32^ | Swine | 70 | LAD embolization | closed | CI < 1.5 L/min/m² and SvO2 - 30% or ≤ 50% | 80 min | unknow |  |  | Impella CP |

BP: Blood Pressure, CO: cardiac output, CS : Cardiogenic Shock, Cx : Circumflex, ECLS: ExtraCorporeal Life Support, FiO2: fraction of Inspirated oxygen, IABP : IntraAortic Balloon Pump conterpulsation, LVEF: Left Ventricular Ejection Fraction, LAD: Left anterior descending artery, LVEDP: Left Ventricular End Diastolic Pressure, MAP: Mean Artery Pressure, NIRS: Near-InfraRed Spectroscopy , SAP: Systolic Artery Pressure, SvO2: Mixed venous oxygen saturation.

Feasibility was defined as the percentage of animals in whom the cardiogenic shock could be obtained. The survival rate was the percentage of animal died during the observation time.

1. Lluch, S. *et al.* *Circulation* **39**, 205–218 (1969).

2. Feola, M. *et al.* *Am Heart J.* **93**, 82–88 (1977).

3. Chandraratna, P. *et al.* *Cardiovascular Research* **7**, 614-622 (1973)

4. Bleifeld, W. *et al.* *Basic Res. Cardiol.* **69**, 379–401 (1974).

5. Freye, E. *et al.* *Resuscitation* **3**, 105–113 (1974).

6. Romero, L. H. *et al. J. Surg. Res.* **16**, 185–191 (1974).

7. Weisse, A. B. *et al.* *Am. Heart J.* **87**, 88–96 (1974).

8. Bavaria, J. E. *et al.* *Ann. Thorac. Surg.* **45**, 526–532 (1988).

9. Wouters, P. F. *et al.* *Eur. Heart J.* **14**, 567–575 (1993).

10. Reilly, P. *et al.* *Gastroenterology* **113**, 938–945 (1997).

11. Jin, X. *et al.* *Crit. Care Med.* **28**, 2415–2419 (2000).

12. Drakos, S. G. *et al.ASAIO J.* **51**, 26–29 (2005).

13. Götberg, M. *et al.* *Resuscitation* **81**, 1190–1196 (2010).

14. How, O.-J. *et al.* *Transl. Res.* **156**, 273–281 (2010).

15. Kawashima, D. *et al.* *ASAIO J. Am. Soc. Artif. Intern. Organs* **57**, 169–176 (2011).

16. Andersson, P. *et al.* *SHOCK* ***37,*** *234-238 (2012).*

17. Brehm, C. *et al. Artif. Organs* **39**, 171–176 (2015).

18. Møller-Helgestad, O. K. *et al. Int J Cardiol.* ***178,*** *153-158 (2015).*

19. Stenberg, T. A. *et al.* *PloS One* **9**, e105213 (2014).

20. Zhu, G. *et al.* *Heart Vessels* **30**, 669–674 (2015).

21. Ostadal, P. *et al.* *J. Transl. Med.* **13**, 266 (2015).

22. Ostadal, P. *et al.* *Physiol. Res.* **65**, 711–715 (2016).

23. Beurton, A. *et al.* *Shock Augusta Ga* **46**, 214–218 (2016).

24. Koudoumas, D. *et al.* *Hell. J. Cardiol. HJC Hell. Kardiologike Epitheorese* **58**, 306–309 (2017).

25. Vanhuyse, F. *et al.* *Shock Augusta Ga* **47**, 236–241 (2017).

26. Trivella, M. G. *et al.* *Int. J. Artif. Organs* **40**, 338–344 (2017).

27. Bari, G. *et al.* *Turk. J. Surg.* **34**, 205–211 (2018).

28. Simonsen, C. *et al.* *Scand. J. Trauma Resusc. Emerg. Med.* **26**, 103 (2018).

29. Ostadal, P. *et al.* *PloS One* **13**, e0196321 (2018).

30. Møller-Helgestad, O. K. *et al.* *EuroIntervention J. Eur.* **14**, e1585–e1592 (2019).

31. Ferrari, M. W. *et al.* *Ther. Adv. Cardiovasc. Dis.* **14**, 1753944719895902 (2020).

32. Udesen, N. L. J. *et al.* *Crit. Care Lond. Engl.* **24**, 95 (2020).
